# Synteny of human chromosomes 14 and 15 in the platyrrhines (Primates, *Platyrrhini*)

**DOI:** 10.1590/S1415-47572009005000069

**Published:** 2009-12-01

**Authors:** Cristiani Gifalli-Iughetti, Célia P. Koiffmann

**Affiliations:** Centro de Estudos do Genoma Humano, Departamento de Genética e Biologia Evolutiva, Instituto de Biociências, Universidade de São Paulo, São Paulo, SPBrazil

**Keywords:** HSA 14/15 synteny, homologies, chromosomal evolution, *Platyrrhini*, New World monkeys

## Abstract

In order to study the intra- and interspecific variability of the 14/15 association in *Platyrrhini*, we analyzed 15 species from 13 genera, including species that had not been described yet. The DNA libraries of human chromosomes 14 and 15 were hybridized to metaphases of *Alouatta guariba clamitans*, *A. caraya*, *A. sara*, *Ateles paniscus chamek*, *Lagothrix lagothricha*, *Brachyteles arachnoides*, *Saguinus midas midas*, *Leontopithecus chrysomelas*, *Callimico goeldii*, *Callithrix sp.*, *Cebus apella*, *Aotus nigriceps*, *Cacajao melanocephalus,Chiropotes satanas* and *Callicebus caligatus*. The 14/15 hybridization pattern was present in 13 species, but not in *Alouatta sara* that showed a 14/15/14 pattern and *Aotus nigriceps* that showed a 15/14/15/14 pattern. In the majority of the species, the HSA 14 homologue retained synteny for the entire chromosome, whereas the HSA 15 homologue displayed fragmented segments. Within primates, the New World monkeys represent the taxon with the highest variability in chromosome number (2n = 16 to 62). The presence of the HSA 14/15 association in all species and subspecies studied herein confirms that this association is the ancestral condition for platyrrhines and that this association has been retained in most platyrrhines, despite the occurrence of extensive inter- and intrachromosomal rearrangements in this infraorder of Primates.

The neighboring association of human chromosome 14 and 15 segments is a well-known ancestral mammalian synteny observed in primates and non-primates and is an important evolutionary landmark (Ruiz-Herrera *et al.*, 2005a). In the majority of cases, the association involves conserved syntenies of the complete human chromosomes (Frönicke, 2005). Therefore, the two human chromosomes clearly represent one ancestral eutherian chromosome (Chowdhary *et al.*, 1998). This association was found in all Zoo-FISH studies except in *Canis familiaris* (*Canidae*, Carnivora) (Breen *et al.*, 1999), *Ailuropoda melanoleuca* (*Ursidae*, Carnivora) (Nash *et al.*, 1998), *Alouatta sara* and *A. arctoidea* (*Atelidae*, *Platyrrhini*, Primates) (Consigliere *et al.*, 1996), and in lesser apes and great apes (Jauch *et al.*, 1992; Haig, 1999; Wienberg, 2005; Ferguson-Smith and Trifonov, 2007).

In all *Platyrrhini* (New World monkeys, Primates) species that have been investigated with whole human chromosome 14 and 15 paints (WCP), conserved segments of the entire HSA (*Homo sapiens*) 14 and fissioning of HSA 15 were observed, although the 14/15 association was still conserved (Seuánez *et al.*, 1994; Consigliere *et al.*, 1996; Richard *et al.*, 1996; Morescalchi *et al.*, 1997; Consigliere *et al.*, 1998; García *et al.*, 1999, 2000; Stanyon *et al.*, 2000; Mudry *et al.*, 2001; Müller *et al.*, 2001; Neusser *et al.*, 2001; Stanyon *et al.*, 2001; De Oliveira *et al.*, 2002; García *et al.*, 2002; Barros *et al.*, 2003; Stanyon *et al.*, 2003; Gerbault-Serreau *et al.*, 2004). Moreover, the 14/15 association was also present in *Saimiri* (Stanyon *et al.*, 2000), *Cebus* (García *et al.*, 2002) and *Aotus* (Stanyon *et al.*, 2004; Ruiz-Herrera *et al.*, 2005b), although it appears to have undergone intrachromosomal reorganizations.

In order to study the intra- and interspecific variability of the 14/15 syntenic association in *Platyrrhini*, we analyzed 15 species from 13 genera and described for the first time the HSA 14/15 association in the species *Ateles paniscus chamek* (*Atelidae*), *Saguinus midas midas* and *Aotus nigriceps* (*Cebidae*), *Cacajao melanocephalus*, *Chiropotes satanas*, and *Callicebus caligatu*s (*Pithecidae*). We also confirmed the association of these chromosomes in nine previously described species of platyrrhines: *Alouatta sara*, *A. guariba clamitans*, *A. caraya*, *Lagothrix lagothricha*, and *Brachyteles arachnoides* (*Atelidae*), *Leontopithecus chrysomelas*, *Callimico goeldii*, *Callithrix sp.*, and *Cebus apella* (*Cebidae*).

Using standard protocols for blood cell and lymphocyte cultures, metaphases were obtained from females of the species *Alouatta guariba clamitans*, *A. caraya*, *Lagothrix lagothricha*, *Saguinus midas midas*, *Leontopithecus chrysomelas*, *Callimico goeldii*, *Aotus nigriceps*, *Chiropotes satanas*, and *Callicebus caligatus* and from males of the species *Alouatta sara*, *Ateles paniscus chamek*, *Brachyteles arachnoides*, *Callithrix sp.*, *Cebus apella*, and *Cacajao melanocephalus.* Chromosomes were prepared and stored in fixative at -20 °C. *Lagothrix lagothricha*, *Ateles paniscus chamek*, and *Cebus apella* are kept at Fundação Parque Zoológico de São Paulo (São Paulo, SP, Brazil); *Saguinus midas midas*, *Leontopithecus chrysomelas*, *Callimico goeldii*, *Aotus nigriceps*, and *Brachyteles arachnoides* at Zoológico Municipal Quinzinho de Barros (Sorocaba, SP, Brazil); *Callicebus caligatus* at Zôo Parque de Itatiba (Itatiba, SP, Brazil); *Cacajao melanocephalus* at Centro de Primatologia do Rio de Janeiro (Rio de Janeiro, RJ, Brazil); *Callithrix sp.* at Barragem Paraitinga (Salesópolis, SP, Brazil); *Chiropotes satanas* and *Alouatta sara* at Fundação Zôo-Botânica de Belo Horizonte (Belo Horizonte, MG, Brazil); *A. guariba clamitans* at Departamento de Parques e Áreas Verdes do Estado de São Paulo, Divisão Técnica de Medicina Veterinária e Manejo da Fauna Silvestre (São Paulo, SP, Brazil); and *A. caraya* at Presidente Epitácio (Porto Primavera, SP, Brazil).

Fluorescent *in situ* hybridization (FISH) with WCP 14 and 15 (Oncor, USA) was carried out according to the manufacturer's protocol. Chromosome preparations were hybridized for two to seven days at 37 °C. Chromosomes were counterstained in blue with DAPI (4,6 diamidino-2-phenylindole) and Vectashield (Switzerland). Metaphase images were obtained with a CCD camera coupled to a Zeiss Axiophot 2 motorized microscope and analyzed with the MetaSystem Isis software (USA).

In the karyotype of one female specimen of *Alouatta guariba clamitans* (2n = 50) from the State of São Paulo, Brazil, the HSA 14 homologue (red) was located in the distal region of the long arm of a large submetacentric pair of chromosomes, juxtaposed to a WCP 15 signal (15a_1_, green). Other signals produced by HSA 15 painting were found in the distal region of the long arm of the typical large submetacentric pair of *A.guaribaclamitans* (15a_2_) and on the short arm of a medium-sized submetacentric pair (15b). De Oliveira *et al.* (2002) described two male specimens from the State of Paraná with 2n = 45, and in both WCP 14 hybridized to the same large submetacentric pair, whereas the human WCP 15 labeled two autosomal pairs (15a_1_ and 15a_2_) and chromosomes X_2_ and Y_1_ (15b) (for males), as in our female.

In *Alouatta caraya* (2n = 52), the HSA 14 homologue (red) was found in the distal region of the long arm of a medium-sized acrocentric pair, juxtaposed to a proximal region painted by WCP 15 (15a_1_, green). The other homologies with HSA 15 were found on the short arm of a medium-sized submetacentric pair (15b, X_2_) and on the long arm of a medium-sized acrocentric pair (15a_2_). A male and a female of *Alouatta caraya* (2n = 52) analyzed by De Oliveira *et al.* (2002) displayed the same hybridized pairs as our specimen, except for a pair of acrocentric chromosomes painted in the proximal region, whereas in our specimen the labeled region (15a_2_) was distal.

In *Alouatta sara* (2n = 50) (Figure 1a), WCP 14 (red) was found to be present along almost the entire length of a medium-sized submetacentric pair, except for the proximal region of the long arm that was painted by WCP 15 (15a_1_, green), showing a 14/15/14 hybridization pattern. Two other pairs were also painted by WCP 15, one a heteromorphic pair (15b, the submetacentric chromosome with the signal across the short arm and the acrocentric chromosome with the signal in the distal region of the long arm), and the other a large acrocentric pair (15a_2_, in the distal region of the long arm). The heteromorphic pair hybridized by WCP 15 corresponds to the sex chromosomes X_2_ (originally a submetacentric autosome) and Y_1_ (a translocation product of the original Y chromosome), resulting from a Y-autosome translocation that is rare in primates, except in *Platyrrhini* (Stanyon *et al.*, 1995; Consigliere *et al.*, 1996). The HSA 14/15 association in *A. sara* (2n = 50) was not observed by Consigliere *et al.* (1996), probably because the signal that identifies the segment homologous to HSA 15, that is located between the WCP 14 signals, is small and difficult to visualize.

**Figure 1:**
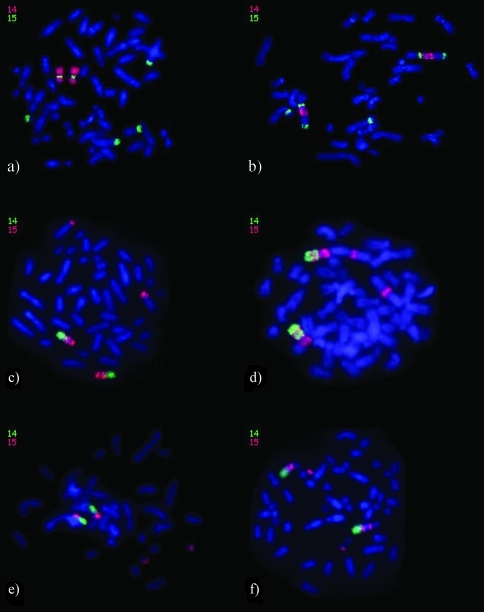
*In situ* hybridization of human chromosome 14 and 15 whole chromosome probes (WCP) on metaphases of: (a) *Alouatta sara*, (b) *Ateles paniscus chamek*, (c) *Saguinus midas midas*, (d) *Aotus nigriceps*, (e) *Cacajao melanocephalus*, and (f) *Chiropotes satanas*. In (a) and (b): WCP 14 - red, WCP 15 - green; in (c-f): WCP 14 - green, WCP 15 - red.

In *Ateles paniscus chamek* (2n = 34), the HSA 14 homologue (red) was located on the long arm of a large submetacentric pair, juxtaposed to a proximal region painted by WCP 15 (15a_1_, green). Another WCP 15 signal was detected in the distal region of the long arm of the same chromosome (15a_2_). A third signal produced by HSA 15 painting was detected in the proximal region of the short arm of a medium-sized submetacentric pair (15b) (Figure 1b). In previous studies, *A. p. paniscus* (2n = 32), *A. belzebuth marginatus* (2n = 34) (De Oliveira *et al.*, 2005), *A. b. hybridus* (2n = 34) (García *et al.*, 2002), and *A. geoffroyi* (2n = 34) (Morescalchi *et al.*, 1997) had shown the same hybridized chromosome pairs as our *A. paniscus chamek* specimen. The drastic reduction in the diploid numbers of *Ateles* (2n = 32, 34), compared to other *Atelidae* genera, has been explained as resulting from several chromosomal rearrangements, including translocations and *in tandem* fusions. These rearrangements also include the HSA 15 homologue, since two WCP 15 signals were detected on a large submetacentric pair (15a_1_ and 15a_2_), whereas these signals were split onto two different chromosomes in other *Atelidae* species.

In *Lagothrix lagothricha* (2n = 62), and *Brachyteles arachnoides* (2n = 62), WCP 14 (red) painted the long arm of a medium-sized submetacentric pair, juxtaposed to a WCP 15 signal (15a_1_, green) on the short arm. Almost the entire long arm of a small acrocentric pair (15b) and the distal region of a medium-sized acrocentric pair also hybridized to WCP 15 (15a_2_). Stanyon *et al.* (2001) studied a female of *Lagothrix lagothricha* (2n = 62) and De Oliveira *et al.* (2005) studied a male of *Brachyteles arachnoides* (2n = 62) and differences in the morphology of the labeled pairs were observed: in *L. lagothricha*, the chromosome pair with the 14/15 association (pair 21) was acrocentric, whereas in *B. arachnoides* it was submetacentric (pair 9). Also in *Lagothrix,* an acrocentric pair was labeled by WCP 15 in the distal region, whereas in *Brachyteles* it was labeled in the proximal region (15a_2_), probably indicating an inversion process.

In the *Atelidae* family species studied herein, the HSA 15 homologue was divided into six blocks located on three chromosome pairs in *Alouatta guariba clamitans*, *A. caraya*, *A. sara*, *Lagothrix lagothricha*, and *Brachyteles arachnoides*, and on two chromosome pairs in *Ateles paniscus chamek*.

In the *Callitrichinae* (*Cebidae*) subfamily species studied herein, *Saguinus midas midas* (2n = 46) (Figure 1c), *Leontopithecus chrysomelas* (2n = 46), *Callithrix sp.* (2n = 46) and *Callimico goeldii* (2n = 48), we observed that WCP 14 (green) was located in the distal region of the long arm of a medium-sized submetacentric pair, while the proximal region of the long arm as well as the entire short arm (except for the centromeric region) were painted by WCP 15 (15a, red). The short arm of a medium-sized submetacentric pair was also painted by WCP 15 (15b), except for *Callimico goeldii* that had the last WCP 15 signal in the proximal region of an acrocentric pair (15b). The *Callimico goeldii* (2n = 47) specimen reported by Neusser *et al.* (2001) also presented the WCP 15 signals in the proximal region of an acrocentric pair (15b) and on the submetacentric pair, associated with WCP 14 (14/15a). In *Saguinus oedipus* (2n = 46) (Müller *et al.*, 2001), *Leontopithecus chrysomelas* (2n = 46) (Gerbault-Serreau *et al.*, 2004), *Callithrix jacchus* (2n = 46) (Sherlock *et al.*, 1996; Neusser *et al.*, 2001), and *C. argentata* (2n = 44) (Neusser *et al.*, 2001), the same submetacentric pairs hybridized to WCP 14 and 15.

In *Cebus apella* (2n = 54) (*Cebidae*), WCP 14 (green) painted the distal region of the long arm of a medium-sized submetacentric pair. The WCP 15 signals (red) were juxtaposed to WCP 14 in the proximal region of the long arm and on the short arm of the same chromosome pair, except in the centromeric region (15a). In the distal region of a small acrocentric pair, we observed another WCP 15 signal (15b). García *et al.* (2000) reported for the same species a 14/15/14 hybridization pattern on a submetacentric pair (pair 6) and a WCP 15 signal on an acrocentric pair. The pattern of the submetacentric chromosome pair could be due to a chromosomal inversion.

In *Aotus nigriceps* (2n = 51) (*Cebidae*), WPC 14 (green) painted the long arm of a medium-sized submetacentric pair, except for a small region painted by WCP 15 (15a_2_) that also hybridized to the short arm of the same chromosome (15a_1_), showing a 15/14/15/14 hybridization pattern. Another WCP 15 signal was located in a region near the centromere on the long arm of the largest submetacentric pair (15b) (Figure 1d). Stanyon *et al.* (2004) and Ruiz-Herrera *et al.* (2005b) studied *A. nancymae* (2n = 54) and found that the paint specific for HSA 14 was split into three segments on two chromosome pairs, whereas the paint specific for HSA 15 was located in six segments of different chromosomes per haploid set; the 14/15 association was found on two chromosomes, and a 14/15/14 hybridization pattern was found on a submetacentric pair (pair 14). In *Aotus sp.,* reported by Ruiz-Herrera *et al.* (2005b) (2n = 50), WCP 14 labeled one pair, whereas WCP 15 was split into six segments of five chromosome pairs, and a 15/14/15/14 hybridization pattern was seen. According to the studies available to date, *Aotus* appears to represent a highly diverse group with several specific chromosomal variations, and with respect to the ancestral New World monkey karyotype, this genus presents a highly derived situation.

In *Cacajao melanocephalus* (2n = 45), WCP 14 (green) hybridized to the long arm of a medium-sized submetacentric pair, while WCP 15 (red) painted the short arm of this chromosome (15a) as well as the distal region of the long arm of a small acrocentric pair (15b) (Figure 1e). The presence of the HSA 14/15 association in the genus *Cacajao* had not been previously reported.

In *Callicebus caligatus* (2n = 48, this work), *C. moloch* (2n = 50) (Stanyon *et al.*, 2000), *C. donacophilus pallescens* (2n = 50) (Barros *et al.*, 2003), and *C. cupreus* (2n = 46) (Dumas *et al.*, 2005), WCP 14 (green) hybridized to the proximal region of an acrocentric pair, whereas WCP 15 labeled the distal region of the same chromosomes (15a) and also the short arm of a medium-sized submetacentric pair (15b). Even in *C. lugens* with its 16 chromosomes (Stanyon *et al.*, 2003), WCP 14 produced a signal on one chromosome pair and WCP 15 on two.

In *Chiropotes satanas* (2n = 54), WCP 14 (green) painted the distal region of the long arm of the largest submetacentric pair. The WCP 15 signals (red) were juxtaposed to WCP 14 (15a_1_) and located in the proximal region of the long arm of the same chromosome (15a_2_). The distance between these two WCP 15 signals was different for the two homologues, probably because of an inversion process. Another WCP 15 signal was identified in the distal region of a small acrocentric pair (15b) (Figure 1f). In *C. utahicki* (2n = 54) and *C. israelita* (2n = 54) (Stanyon *et al.*, 2004), the same painting pattern had been observed, and they apparently showed the same hybridized chromosome pairs as *C. satanas*, except for the large submetacentric pair that displayed a single block homologous to HSA 15.

In the *Pithecidae* family species studied herein, we observed two chromosomes hybridized by WCP 15, showing four signals in *Cacajao melanocephalus* and *Callicebus caligatus*, and six signals in *Chiropotes satanas*, due to the presence of two separate signals on the same chromosome arm.

Table 1 summarizes the results obtained by hybridizing human WCP 14 and 15 to the chromosomes of the species we analyzed and to those that have been described in literature.

Within primates, the *Platyrrhini* infraorder represents the group with the greatest variability in chromosome number, ranging from 2n = 16 in *Callicebus lugens* to 2n = 62 in *Brachyteles* and *Lagothrix*. A 14/15 hybridization pattern was observed in 13 species of 13 genera, the exceptions being *Alouatta sara*, which showed a 14/15/14 pattern, and *Aotus nigriceps*, which showed a 15/14/15/14 pattern. In all of the species, the HSA 14 homologue retained synteny for the entire chromosome, except for *A. nancymae*, whereas the HSA 15 homologue underwent fragmentations, leading to a shared derived fission of the ancestral mammalian 14/15 form to 14/15a and 15b in the ancestral *Platyrrhini* karyotype.

Despite the occurrence of extensive inter- and intrachromosomal rearrangements within the platyrrhines, the presence of a HSA 14/15 syntenic association in all species and subspecies studied so far confirms that this karyotypic feature is the ancestral condition of this group of primates. Furthermore, its presence in platyrrhines appears to be the retention of the pattern presumed to be the primitive mammalian pattern.

The HSA 14/15 syntenic association is present in all primates except in the lineage leading to apes. Chromosome fission after the divergence of Old World monkeys has led to the separation of HSA 14 and 15 (Haig, 1999; Wienberg, 2005; Ferguson-Smith and Trifonov, 2007).

In humans, chromosome 15 shows a high degree of genomic instabilities, particularly in the proximal region, which has been found to be associated with various genomic diseases, as a consequence of deletions, duplications, translocations and inter- and intrachromosomal rearrangements in this region.

## Figures and Tables

**Table 1:** Summarized results of WCP 14/15 hybridization in *Platyrrhini* observed in this work and in the literature.

Genera	Species			WCP 14/15						WCP 15			References
	this work	literature		submeta			acro		submeta			acro	
				p	q				p	q			
*Alouatta*	*guariba clamitans*				+dist				+	+dist			
		*guariba clamitans*			+dist				+	+dist			De Oliveira *et al.* (2002)
	*caraya*						+		+			+dist	
		*caraya*					+		+			+prox	De Oliveira *et al.* (2002)
	*sara*			+	+				±			±dist, +dist	
		*sara*		¤	¤				+prox, ±	+prox, ±		+dist	Consigliere *et al.* (1996)
*Ateles*	*paniscus chamek*				+prox				+prox	+dist			
		*paniscus paniscus*			+prox				+prox	+dist			De Oliveira *et al.* (2005)
		*belzebuth marginatus*			+prox				+prox	+dist			De Oliveira *et al.* (2005)
		*belzebuth hybridus*			+prox				+prox	+dist			García *et al.* (2002)
		*geoffroyi*			+prox				+prox	+dist			Morescalchi *et al.* (1997)
*Lagothrix*	*lagothricha*			+	+							+, +dist	
		*lagothricha*					+					+, +dist	Stanyon *et al.* (2001)
*Brachyteles*	*arachnoides*			+	+							+, +dist	
		*arachnoides*		+	+							+, +prox	De Oliveira *et al.* (2005)
*Saguinus*	*midas midas*			+	+				+				
		*oedipus*		+	+				+				Müller *et al.* (2001)
*Leontopithecus*	*chrysomelas*			+	+				+				
		*chrysomelas*		+	+				+				Gerbault-Serreau *et al.* (2004)
*Callithrix*	*sp.*			+	+				+				
		*jacchus*		+	+				+				Sherlock *et al.* (1996), Neusser *et al.* (2001)
		*argentata*		+	+				+				Neusser *et al.* (2001)
*Callimico*	*goeldii*			+	+							+prox	Neusser *et al.* (2001)
		*goeldii*		+	+							+prox	
*Cebus*	*apella*			+	+							+dist	
		*apella*		+	+							+	García *et al.* (2000)
*Aotus*	*nigriceps*			+	+					+prox			
		*nancymae*		+	+, +				+prox	+prox		+prox, +dist	Stanyon *et al.* (2004), Ruiz-Herrera *et al.* (2005b)
		*sp.*					+			+prox		+prox, +dist, +dist	Ruiz-Herrera *et al.* (2005b)
*Cacajao*	*melanocephalus*			+	+							+dist	
*Chiropotes*	*satanas*				+dist, prox							+dist	
		*utahicki*			+							+	Stanyon *et al.* (2004)
		*israelita*			+							+	Stanyon *et al.* (2004)
*Callicebus*	*caligatus*						+		+				
		*moloch*					+		+				Stanyon *et al.* (2000)
		*donacophilus pallescens*					+		+				Barros *et al.* (2003)
		*cupreus*					+		+				Dumas *et al.* (2005)

submeta = submetacentric chromosome. acro = acrocentric chromosome. p = short arm. q = long arm. + = presence of the signal. dist = distal region. prox = proximal region. ± = heteromorphic pair. ¤ = only WCP 14 signal; the 14/15 association was not found.
